# Effects of Modified Thoracoabdominal Nerves Block Through Pericondrial Approach on Postoperative Pulmonary Functions in Laparoscopic Bariatric Surgery: A Randomized Controlled Study

**DOI:** 10.1007/s11695-025-07908-3

**Published:** 2025-05-21

**Authors:** Çağdaş Baytar, Zeynep Gürbüz, Bengü Gülhan Köksal İncegül, Merve Sena Baytar, İlhan Taşdöven, Özcan Pişkin

**Affiliations:** 1 Zonguldak Bülent Ecevit University Medicine Faculty, Department of Anesthesiology and Reanimation, Zonguldak, Turkey; 2 Zonguldak Bülent Ecevit University Medicine Faculty, Department of General Surgery, Zonguldak, Türkiye

**Keywords:** Obesity, Laparoscopic sleeve gastrectomy, Modified thoracoabdominal nerves block through pericondrial approach, Respiratory dysfunction

## Abstract

**Background:**

We aimed to evaluate the effects of modified thoracoabdominal nerve block through perichondrial approach (M-TAPA) block on respiratory dysfunction after laparoscopic sleeve gastrectomy (LSG) in patients with obesity.

**Methods:**

In this prospective randomized-controlled study, 60 patients aged 18–65 years and ASA PS II-III were included. Patients were divided into two groups: group M-TAPA (*n* = 30) and group control (*n* = 30). The primary outcome was the results of spirometric respiratory function tests. The secondary outcomes were total opioid consumption, postoperative resting, dynamic pain scores, and assessing the functional recovery via quality of recovery (QoR)-15 on postoperative day 1.

**Results:**

The FVC, FEV1, PEF, and predicted FEV1 values were significantly different between the groups, whereas the results were similar for FEV1/FVC values in the postoperative first hour. The decreases in FVC, FEV1, PEF, and predicted FEV1 values were higher in the group control. Total tramadol consumption at 0–24 h was significantly higher in the group control than in the group M-TAPA (group M-TAPA = 170 (0–300), group control = 220 (80–300); *p* = 0.013). Resting and dynamic NRS pain scores at the 1st, 2nd, 6th, and 12th postoperative hours were significantly higher in the control group than in the M-TAPA group.

**Conclusions:**

The M-TAPA block in patients with obesity undergoing LSG reduced postoperative respiratory dysfunction and opioid consumption.

## Introduction

Obesity is on the rise and is a major health concern in almost every country in the world. Laparoscopic sleeve gastrectomy (LSG) is increasingly used to treat obesity and has shown successful outcomes [1, 2]. However, anesthetic management of these patients is challenging, and LSG is associated with a risk of mortality and morbidity [2].

Increased abdominal volume and visceral fat in patients with obesity negatively affect respiratory function. Lung volume and respiratory system compliance decrease, while airway resistance and breathing effort increase, leading to deteriorated spirometric values [3].

Ventilatory dysfunction commonly develops after abdominal surgery and may persist for up to 10 days postoperatively due to muscle trauma, diaphragmatic reflex inhibition, and pain [4]. Abdominal surgery further worsens pulmonary mechanics in patients with obesity, whose respiratory function is already compromised. This increases the risk of postoperative pulmonary complications, morbidity, and mortality [3].

Effective perioperative pain management has been shown to reduce respiratory complications, particularly after upper abdominal surgery [5]. Modified thoracoabdominal nerves block through pericondrial approach (M-TAPA) is the application of local anesthetic between the transversus abdominis muscle and the costal cartilage from the midclavicular line at the level of the 10 th costal cartilage [6]. The M-TAPA block, which targets the anterior and lateral branches of the thoracoabdominal nerves, provides effective analgesia during LSG [7].

We aimed to evaluate the effects of the M-TAPA block on respiratory dysfunction after LSG in patients with obesity, whose pulmonary mechanics are already compromised. The primary outcome of this study was the assessment of spirometric respiratory function test results. The secondary outcomes were total opioid consumption, postoperative resting, and dynamic pain scores, and assessing the functional recovery via quality of recovery (QoR)−15 on postoperative day 1.

## Methods

### Study Design

This study was conducted as a prospective randomized controlled study in Zonguldak Bülent Ecevit University Hospital between December 2023 and May 2024. The study was approved by the Local Ethics Committee (protocol number: 2023/04–4, ClinicalTrials.gov identifier: NCT06148597). All patients were informed in detail about the study, and their verbal and written consent was obtained. This study was conducted according to the CONSORT guidelines.

### Study Population

Sixty patients aged 18–65 years with an American Society of Anesthesiologists Physical Status (ASA PS) of 2–3 who underwent elective LSG under general anesthesia were included in the study. Exclusion criteria included refusal to participate, ASA PS 4–5, allergy to local anesthetics, coagulopathy, smoking, infection at the block site, chronic analgesic use, inability to cooperate, spirometric results below 50% of expected values, known diaphragmatic paralysis, dementia or confusion, respiratory disease, congestive heart failure, unstable hypertension, severe renal or liver disease, previous thoracoabdominal surgery, respiratory tract infection within the past month, and conversion to open surgery.

### Randomization and Blinding

Patients were randomized into two groups using a computer-assisted program and the closed-envelope method: Group M-TAPA (*n* = 30) and group control (*n* = 30). Randomization and sealed envelopes were prepared by a non-study staff member. All assessments and data collection on the ward were carried out by the same non-study staff, who were blind to which group the patient was in. Additionally, the nurses in the ward did not have any information about the randomization. All block applications were performed by the same anesthesiologist (ÇB).

### Preoperative Procedures

All patients were briefed about the numerical rating scale (NRS) score, the QoR-15 questionnaire, and the patient-controlled analgesia (PCA) device and its handling. All patients fulfilled the QoR-15 questionnaire in the premedication room. Then, the pulmonary function of the patients was assessed using spirometry (CONTEC SP10 Digital Spirometer, CONTEC, Hebei, China). First, the patient’s sex, age, height, weight, and smoking status were recorded on the device. A disposable, filtered spirometer mouthpiece was attached to the device. The patient’s nose was clamped using a nose clip, and normal breathing was performed. After that, a deep and powerful breath was taken. With the command, the patient was made to exhale quickly and forcefully for at least 6 s without waiting. After exhaling, deep breathing was retaken, and the test was terminated. Three consecutive measurements were performed in the sitting position. Forced vital capacity (FVC), forced expiratory volume in the first second (FEV1), FEV1/FVC ratio, peak expiratory flow (PEF), and predicted FEV1 values were recorded. The best values from the three measurements were recorded.

### Anesthesia and Analgesia Management

All patients were taken to the operating room, and in addition to routine monitoring, train-of-four (TOF; TOF-Watch SX), bispectral index monitoring (BIS), and pleth variability index (PVI; Masimo-Radical-7™ Pulse CO-Oximeter®) monitoring were applied. All patients received general anesthesia. During induction, they were placed in the ramp position for optimal airway alignment and intubated using intravenous (IV) 1 mg/kg lidocaine, 2–3 mg/kg propofol, 1 mcg/kg fentanyl, and 0.6 mg/kg rocuronium, dosed according to lean body weight. Following intubation, oxygenation of the patients was achieved with a 50–50% air-oxygen mixture. Ventilation was adjusted in a volume-controlled mode according to ideal body weight with a tidal volume of 6–8 mL/kg, PEEP 5–10 cm H_2_O, and a respiratory rate adjusted to maintain EtCO₂ between 35 and 45 mmHg.

**Fig. 1:**
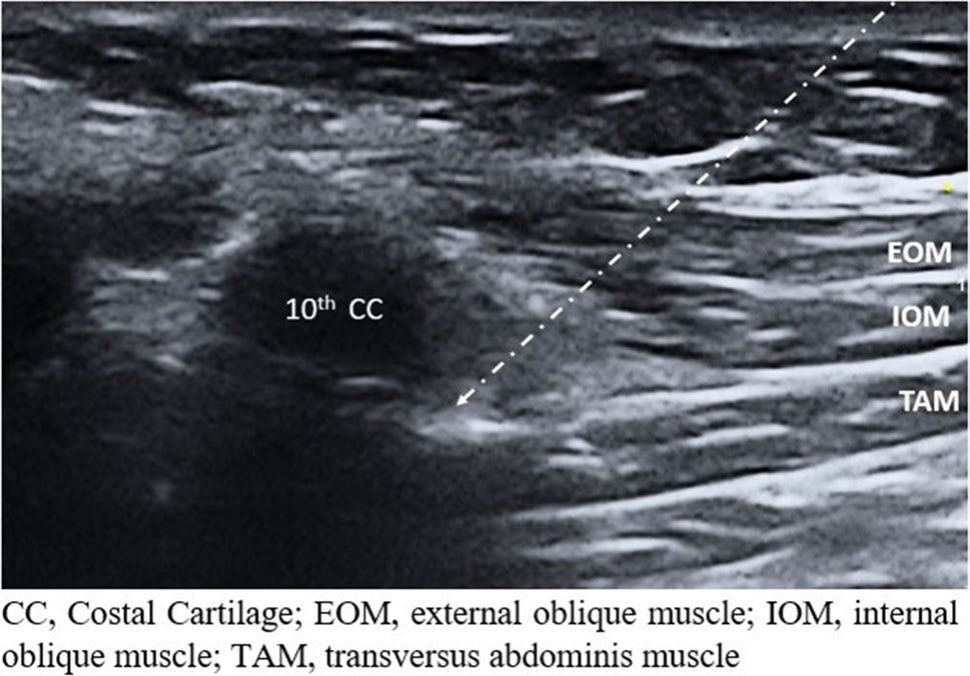
Ultrasound image of M-TAPA block application

After intubation, patients in the M-TAPA group were administered bilateral M-TAPA block in the supine position. After providing the required sterilization, a convex ultrasound transducer (1–8 mHz, Esaote MyLab X7, Italy) was placed on the 10 th costal cartilage on the inferior aspect of the costal margin in the midclavicular line in the sagittal plane. The 10 th costal cartilage, external and internal oblique muscles, and transversus abdominis muscles were identified by tilting the probe cranially. The 80-mm needle was advanced between the transversus abdominis muscle and costal cartilage with an in-plane technique. After confirming the location with 1 mL isotonic saline following negative aspiration, 20 mL of 0.25% bupivacaine was injected with intermittent aspiration (Fig. 1). And then, patients were handed over to the surgical team.

Anesthesia maintenance was provided with sevoflurane with a BIS between 40 and 60 during surgery. Intraoperatively, the patient’s fluid therapy was maintained at a PVI of 14 to 16%. Remifentanil was infused at 0.05–0.2 mcg/kg/min, adjusted based on a 20% change in baseline mean blood pressure. Hemodynamic data were recorded at certain intervals during the intraoperative period. One gram of paracetamol, 100 mg of tramadol, and 10 mg of metoclopramide were administered intravenously 30 min before the end of the surgery. Remifentanil was stopped with the final suture, and the total amount consumed was recorded. The time between the last suture and extubation was defined as emergence time and recorded. At the end of the surgery, all patients were extubated using sugammadex according to adjusted body weight. The duration of anesthesia and surgery were recorded. Patients were transferred to the recovery unit.

### Surgical Technique

All surgeries were performed by a single surgeon using the same technique (IT). A 12-mm trocar was inserted through the periumbilical area while the patient was in the supine position. The abdominal cavity was expanded by insufflating CO_2_ to achieve an intra-abdominal pressure of 12–14 mmHg. The procedures were performed using four trocars in total. A 12-mm trocar was placed under the subcostal area in the right midclavicular line, a 5-mm trocar under the subcostal area in the left midclavicular line, and a 5-mm trocar in the subxiphoid region for liver retraction. The trocar in the periumbilical region was used for the camera. After trocar placement, the patient was positioned in reverse Trendelenburg with a slight incline while in the lithotomy position. At the end of the procedure, a drain was inserted through the 5-mm trocar on the left.

### Postoperative Procedures

In the recovery unit, patients were monitored and received PCA. The PCA device was prepared with tramadol, set to no basal infusion, a 10-mg bolus dose, and a 20-min lockout time. Arrival in the recovery room was defined as hour 0. Resting and dynamic (coughing) NRS pain scores and the level of sedation by using the Ramsey sedation scale (RSS) score were evaluated at the 20 th minute after coming to the recovery room. If the NRS pain score was ≥ 4, 25 mcg IV fentanyl was administered as a rescue analgesic. The pain score was reassessed 15 min later. If the NRS pain score was still ≥ 4, fentanyl was repeated. The total amount of rescue analgesic administered until the patient left the recovery unit was recorded. Any complications, including hypotension, bradycardia, respiratory depression, pruritus, and shoulder pain, were recorded in the recovery unit. The presence of nausea and vomiting was evaluated using a Likert scale (0: none, 1: mild, 2: moderate, 3: severe, 4: vomiting). If the score was ≥ 3, 4 mg IV ondansetron was administered. Data were recorded. Patients were transferred to the ward when the modified Aldrete score was ≥ 9.

In the ward, spirometric PFT was repeated at 1, 6, and 24 h postoperatively. The measurements were repeated three times, and the best values were recorded. FVC, FEV1, FEV1/FVC ratio, PEF, and Predicted FEV1 values were recorded.

Postoperatively, 1 g paracetamol was administered every 8 h. Resting and dynamic NRS pain scores of the patients at 1, 2, 6, 12, and 24 h postoperatively were evaluated and recorded. The RSS scores and whether any complications developed were recorded each time the patients were visited. If there was uncontrolled pain (NRS score ≥ 4) despite PCA and paracetamol in the ward, patients were planned to receive IM 75 mg diclofenac sodium. If the pain persisted after IM injection, patients were planned to receive IV 0.25 mg/kg pethidine according to lean body weight. The amount of rescue analgesic given postoperatively was recorded. The QoR-15 questionnaire was filled out again at the 24 th hour postoperatively. The PCA device was terminated. The amount of opioids consumed by the patient for 24 h, the number of requests, and the number of boluses given were recorded.

### Statistical Analysis

The approximate sample size was calculated using the G*Power program before the start of the study enrolling 10 patients from each group who were not included in the study. The analysis was based on the mean difference between preoperative and 24-h postoperative FEV1 values (M-TAPA group: 1.21 ± 0.45; control group: 1.55 ± 0.55; effect size *d*: 0.67). In the analysis performed using a 95% confidence interval and 80% power, a minimum of 28 patients per group was obtained. The approximate sample size was also calculated based on secondary outcomes. Based on the calculations of total opioid consumption over 24 h (group M-TAPA: 162.00 ± 95.66; group control: 241.00 ± 51.30, effect size (*d*): 1.036) and the global QoR-15 score at postoperative 24 h (group M-TAPA: 114.40 ± 26.43; group control: 87.80 ± 14.55, effect size (*d*): 1.293), using a 95% confidence interval and 80% power, the minimum required sample size per group was determined to be 13 and 9 patients, respectively. A total of 66 patients were included in the study, considering possible missing patients. Data were analyzed using SPSS version 25.0. Descriptive variables are expressed as mean ± standard deviation (SD), median, minimum–maximum (min–max) for quantitative data, and frequency for qualitative data. The Kolmogorov–Smirnov test was used to assess the conformity of quantitative values to the normal distribution in the groups. The *t*-test and Mann–Whitney *U* test were used for analyses. The chi-squared test was used to compare qualitative data between the groups. Results were assessed using 95% confidence intervals (CIs), with *p* < 0.05 considered statistically significant.

## Results

**Fig. 2:**
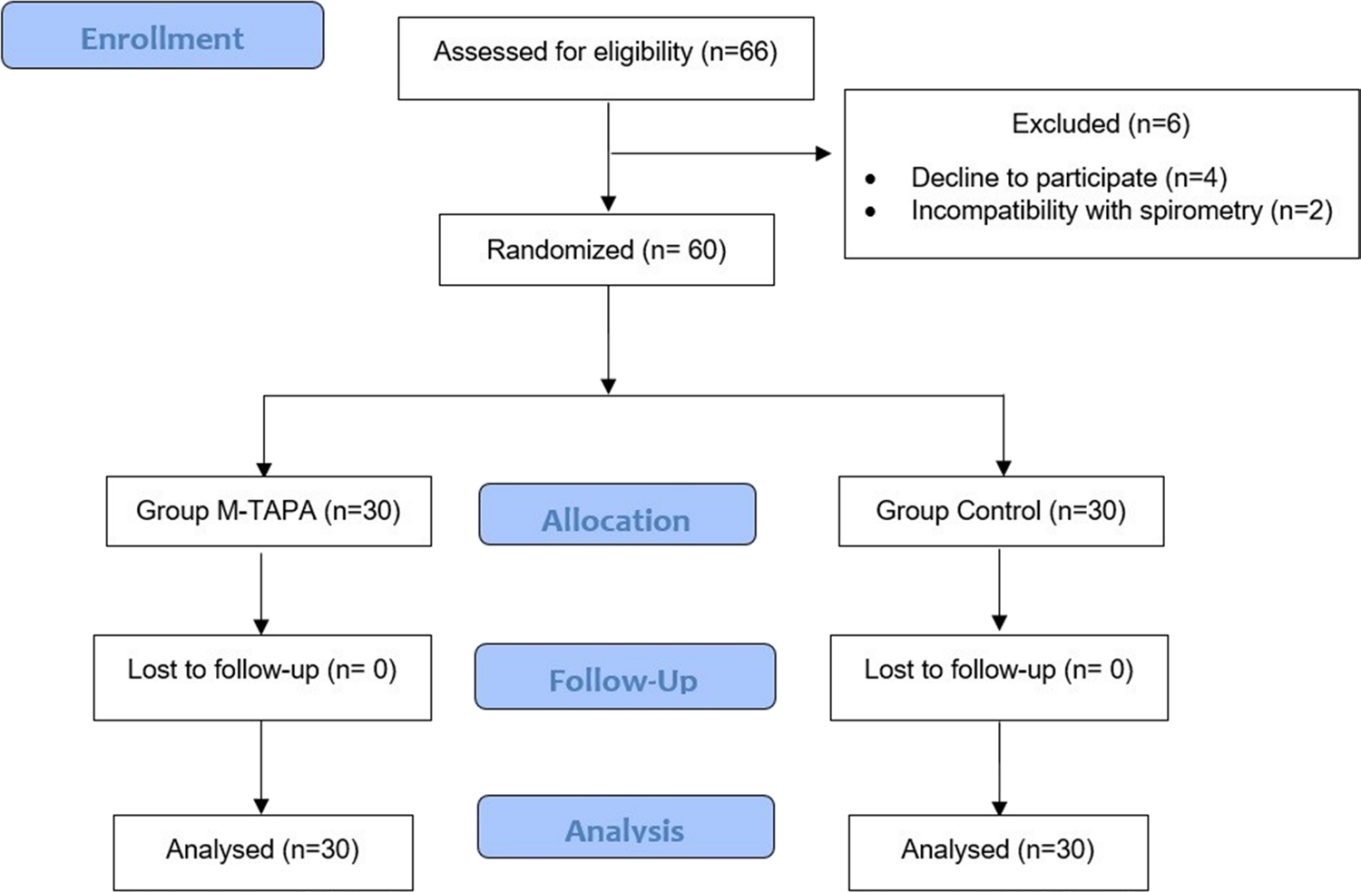
CONSORT flow diagram

The study included 66 patients who underwent LSG. After four patients refused to participate in the study and two patients were unable to perform spirometry, 60 patients were statistically analyzed (Fig. 2). There were no differences in demographic and surgical data between the groups (Table 1). Intraoperative remifentanil consumption was significantly lower in the M-TAPA group (515.00 ± 242.1 µg) than in the control group (1051.67 ± 456.84 µg; *p* < 0.001). Intraoperative hemodynamic parameters, BIS, and PVI values did not differ significantly between groups.

**Table 1:** Demographic and surgical data

	Group M-TAPA (*n* = 30)	Group control (*n* = 30)	*P*
Age (years)^#^	34.80 ± 9.37	34.53 ± 10.34	0.917
Sex (male/female)*	9/21	5/25	0.222
BMI^#^	46.66 ± 5.39	48.57 ± 6.27	0.211
ASA PS (II/III)*	1/29	1/29	1.000
Duration of surgery (min)^#^	39.10 ± 7.95	42.17 ± 10.01	0.395
Duration of anesthesia (min)^#^	78.13 ± 10.58	73.20 ± 12.02	0.990
Emergence time (min)^#^	9.50 ± 2.99	8.47 ± 3.28	0.122
Intraoperative remifentanil consumption (µg)^#^	515.00 ± 242.17	1051.67 ± 456.84	** < 0.001**^**¥**^

*ASA PS*, American Society of Anesthesiologists Physical Status; *BMI,* body mass index, *M-TAPA*, modified thoracoabdominal nerves block through perichondrial approach.^#^: mean ± standard deviation; *: n; ¥: *p* < 0.05

RSS scores at 20 min in the recovery unit were significantly different between groups (M-TAPA: I/II/III = 7/18/5; control: I/II/III = 19/9/2; *p* = 0.007). Resting and dynamic NRS pain scores in the recovery unit were significantly different between groups (group M-TAPA, resting NRS 3 (0–5), dynamic NRS 3 (0–6); group control, resting NRS 5 (2–8), dynamic NRS 6 (3–9); both, *p* < 0.001). A significant difference was found between the groups in terms of fentanyl use in the recovery unit (*p* < 0.001). Fentanyl was administered to 25 patients in the control group and to 5 patients in the M-TAPA group (group M-TAPA, 0 (0–50) µg, group control, 50 (0–75) µg; *p* < 0.001). Vital parameters were similar between the groups in the recovery unit (*p* = 0.07). Assessment of nausea and vomiting in the recovery unit showed no difference between the groups (*p* = 0.118). Any complications did not develop in the recovery unit.

When the results of spirometric PFTs were analyzed, preoperative FVC, FEV1, FEV1/FVC, PEF, and predicted FEV1 values were not significantly different between the groups. In the spirometry repeated at the 1 st hour postoperatively, FVC, FEV1, PEF, and predicted FEV1 values were significantly different between the groups, whereas the results were similar for FEV1/FVC values. The decreases in FVC, FEV1, PEF, and predicted FEV1 values were higher in the group control. In the postoperative 6 th and 24 th hour measurements, the results were similar between the groups for all parameters (Table 2). The comparisons of the 95% confidence intervals for FVC, FEV1, and PEF values by groups are presented in Table 3.

**Table 2:** Results of spirometric respiratory function tests

		Group M-TAPA (*n* = 30)	Group control (*n* = 30)	*P*
FVC, L	Preoperative values	3.48 ± 0.74	3.42 ± 0.70	0.781
	1 h after operation values	2.54 ± 0.73	1.75 ± 0.67	< 0.001 ^*^
	6 h after operation values	2.57 ± 0.74	2.22 ± 0.74	0.139
	24 h after operation values	2.38 ± 0.60	2.20 ± 0.66	0.273
	Preoperative vs. postoperative first hour (mean difference, %)	0.94 (27%)	1.67 (48%)	
	Preoperative vs. postoperative sixth hour (mean difference, %)	1.01 (29%)	1.20 (35%)	
	Preoperative vs. postoperative 24 th hour (mean difference, %)	1.10 (31%)	1.22 (35%)	
FEV1, L	Preoperative values	2.90 ± 0.63	2.84 ± 0.59	0.696
	1 h after operation values	1.98 ± 0.69	1.36 ± 0.58	< 0.001^*^
	6 h after operation values	1.99 ± 0.61	1.75 ± 0.65	0.153
	24 h after operation values	1.87 ± 0.58	1.76 ± 0.52	0.466
	Preoperative vs. postoperative first hour (mean difference, %)	0.92 (31%)	1.48 (52%)	
	Preoperative vs. postoperative sixth hour (mean difference, %)	0.91 (31%)	1.09 (38%)	
	Preoperative vs. postoperative 24 th hour (mean difference, %)	1.03 (35%)	1.08 (38%)	
Predicted FEV1, %	Preoperative values	91 ± 12	92 ± 12	0.710
	1 h after operation values	61 ± 18	44 ± 13	< 0.001^*^
	6 h after operation values	64 ± 17	58 ± 18	0.264
	24 h after operation values	59 ± 17	57 ± 16	0.631
	Preoperative vs. postoperative first hour (mean difference, %)	30 (32%)	48 (%52)	
	Preoperative vs. postoperative sixth hour (mean difference, %)	27 (29%)	34 (36%)	
	Preoperative vs. postoperative 24 th hour (mean difference, %)	32 (35%)	35 (38%)	
FEV1/FVC, %	Preoperative values	84 ± 4	83 ± 4	0.758
	1 h after operation values	78 ± 7	76 ± 7	0.698
	6 h after operation values	77 ± 6	79 ± 6	0.307
	24 h after operation values	78 ± 7	79 ± 7	0.328
	Preoperative vs. postoperative first hour (mean difference, %)	6 (7%)	7 (8%)	
	Preoperative vs. postoperative sixth hour (mean difference, %)	7 (8%)	4 (4%)	
	Preoperative vs. postoperative 24 th hour (mean difference, %)	6 (7%)	4 (4%)	
PEF, L/s	Preoperative values	5.94 ± 1.52	5.76 ± 1.78	0.672
	1 h after operation values	3.39 ± 1.43	2.23 ± 1.12	< 0.001^*^
	6 h after operation values	3.31 ± 1.30	2.97 ± 1.20	0.243
	24 h after operation values	3.37 ± 1.41	3.12 ± 1.11	0.453
	Preoperative vs. postoperative first hour (mean difference, %)	2.55 (42%)	3.53 (61%)	
	Preoperative vs. postoperative sixth hour (mean difference, %)	2.63 (44%)	2.79 (48%)	
	Preoperative vs. postoperative 24 th hour (mean difference, %)	2.57 (43%)	2.64 (45%)	

Data are given as mean ± SD. *M-TAPA*, modified thoracoabdominal nerves block through perichondrial approach; *FVC*, forced vital capacity, *FEV1*, forced expiratory volume in the first second; *PEF*, Peak expiratory flow. *p < 0.05

**Table 3:** Confidence intervals of spirometric respiratory function tests results

		Group M-TAPA (*n* = 30)	Group control (*n* = 30)
FVC, L	Preoperative values 1 h after operation values 6 h after operation values 24 h after operation values	3.20–3.75 2.26–2.81 2.29–2.85 2.16–2.61	3.16–3.65 1.50–2.00 1.54–2.50 1.95–2.45
FEV1, L	Preoperative values 1 h after operation values 6 h after operation values 24 h after operation values	2.66–3.14 1.72–2.24 1.76–2.22 1.65–2.09	2.62–3.06 1.14–1.58 1.51–2.00 1.56–1.96
PEF, L/s	Preoperative values 1 h after operation values 6 h after operation values 24 h after operation values	5.37–6.51 2.85–3.93 2.82–3.80 2.84–3.89	5.09–6.43 1.81–2.65 2.51–3.42 2.70–3.53

Data are difference in means (95%CI). *M-TAPA*, modified thoracoabdominal nerves block through perichondrial approach; *FVC*, forced vital capacity; *FEV1*, forced expiratory volume in the first second; *PEF*, peak expiratory flow

Total tramadol consumption at 0–1 h, 0–2 h, 0–6 h, 0–12 h, and 0–24 h was significantly higher in the group control than in the group M-TAPA (*p* = 0.001, *p* = 0.016, *p* = 0.029, *p* = 0.003, and *p* = 0.013, respectively) (Table 4).

**Table 4:** Cumulative tramadol consumption (Mg)

	Group M-TAPA (*n* = 30)	Group control (*n* = 30)	*P*
1 st hour^#^	20 (0–30)	30 (0–30)	0.001*
2nd hour^#^	40 (0–60)	50 (0–60)	0.016*
6 th hour^#^	90 (0–180)	110 (40–180)	0.029*
12 th hour^#^	110 (0–240)	160 (70–300)	0.003*
24 th hour^#^	170 (0–300)	220 (80–300)	0.013*

*M-TAPA*, modified thoracoabdominal nerves block through perichondrial approach, ^#^: Mean ± standard deviation, *: *p* < 0.05

**Fig. 3:**
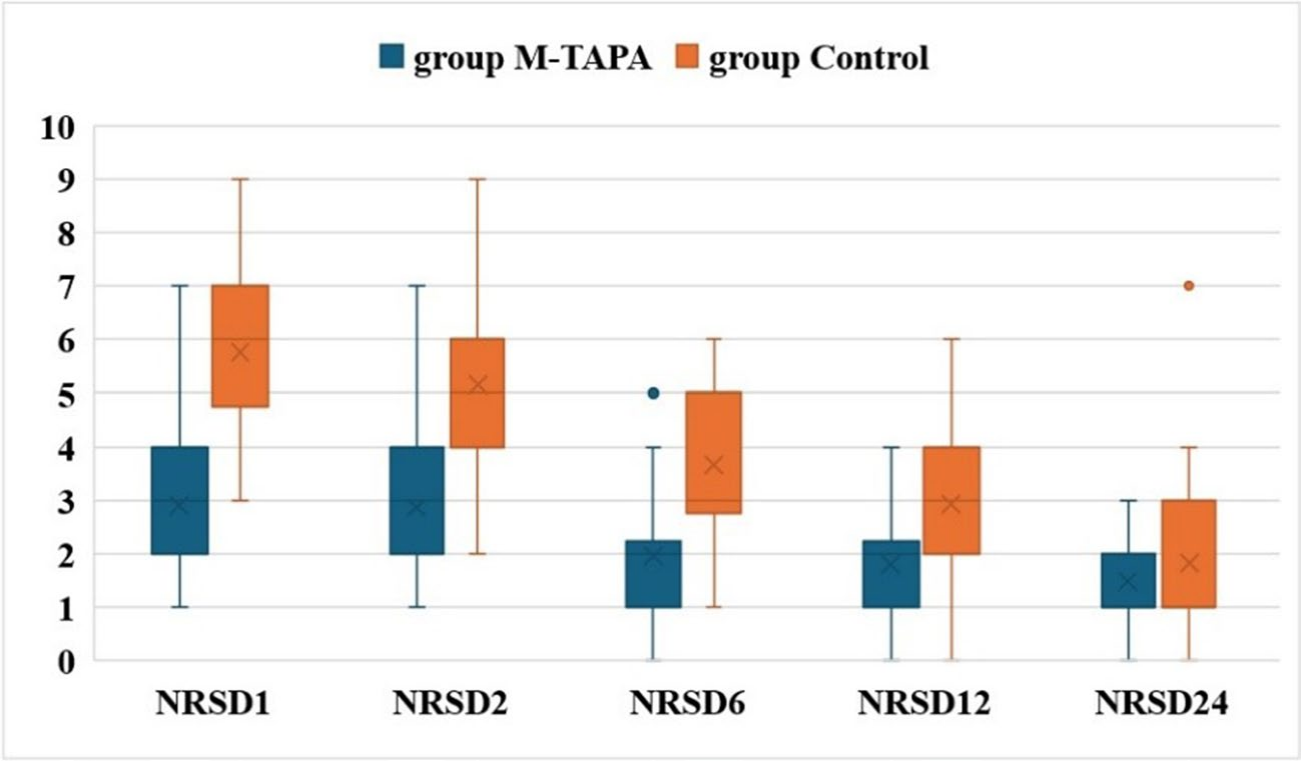
Comparison of the dynamic numerical rating scale pain scores

Both resting and dynamic NRS pain scores evaluated at postoperative 1 st, 2nd, 6 th, and 12 th hours were significantly higher in the group control group than in the group M-TAPA. At the 24 th hour, there was no difference between the groups (Table 5, Fig. 3).

**Table 5:** Comparison of the numerical rating scale pain scores

	NRS_R_/NRS_D_^#^	Group M-TAPA (*n* = 30)	Group control (*n* = 30)	*P*	Effect size
1 st hour	NRS_R_ NRS_D_	2 (0–6) 3 (1–7)	5 (2–8) 6 (3–9)	< 0.001* < 0.001*	** − 0.771** ** − 0.812**
2nd hour	NRS_R_ NRS_D_	2 (0–6) 3 (1–7)	4 (1–8) 5 (2–9)	< 0.001* < 0.001*	** − 0.630** ** − 0.730**
6 th hour	NRS_R_ NRS_D_	1 (0–4) 2 (0–5)	3 (1–5) 3 (1–6)	< 0.001* < 0.001*	** − 0.681** ** − 0.656**
12 th hour	NRS_R_ NRS_D_	1 (0–3) 2 (0–4)	2 (0–5) 3 (0–6)	< 0.001* 0.001*	** − 0.517** ** − 0.464**
24 th hour	NRS_R_ NRS_D_	1 (0–2) 1 (0–3)	0.50 (0–6) 1 (0–7)	0.551 0.620	** − **0.082 ** − **0.070

*NRS*_*R*_, resting numerical rating scale; *NRS*_*D*_, dynamic numerical rating scale; *M-TAPA*, modified thoracoabdominal nerves block through perichondrial approach. ^#^: Median (min–max), *p < 0.05

Preoperative QoR-15 global scores were similar between the groups (*p* = 153). In the QoR-15 questionnaire repeated at the 24 th hour postoperatively, significantly higher scores were obtained in the group M-TAPA compared to the group control (< 0.001). Patients in the group M-TAPA had significantly higher scores in the pain, physical comfort, physical independence, and emotional status subdimensions in the QoR-15 repeated at the 24 h postoperatively. In the psychological support subdimension, the results were similar between the groups (Table 6).

**Table 6:** Quality of recovery-15 scores between patients with and without M-TAPA

QoR-15 score*	Group M_TAPA (*n* = 30)	Group control (*n* = 30)	*P*	Effect size
Before surgery	141 (129–150)	138.50 (119–149)	0.153	
Pain	20 (19–20)	20 (19–20)	0.557	
Physical comfort	46 (33–50)	45 (34–50)	0.389	
Physical independence	20 (16–20)	20 (13–20)	0.626	
Psychological support	20 (17–20)	20 (19–20)	0.388	
Emotional state	37 (24–40)	33 (23–40)	0.054	
24 h after surgery	121 (70–148)	97 (32–126)	< 0.001*	**0.552**
Pain	17 (2–20)	10.50 (2–18)	< 0.001*	**0.607**
Physical comfort	35.50 (17–50)	29 (11–40)	0.001*	**0.472**
Physical independence	13 (3–20)	9 (0–17)	0.005*	**0.402**
Psychological support	19.50 (11–20)	18 (5–20)	0.354	0.144
Emotional state	35 (18–40)	29 (6–40)	0.014*	**0.367**

*A 11-point numerical rating scale (0 for never true and 10 for always true for positive items and vice versa for negative items) applied for each of total 15 items. *M-TAPA*, modified thoracoabdominal nerves block through perichondrial approach; *QoR*, quality of recovery. *p < 0.05

The RSS scores evaluated at all time points in the ward did not differ between the groups. No complication developed within 24 h postoperatively. In postoperative evaluations, the need for IM diclofenac sodium as a rescue analgesic at the 1 st hour was higher in group control (group M-TAPA, *n* = 1, group control, *n* = 8; *p* = 0.011), while there was no significant difference at all other time points. None of the patients in both groups required pethidine. PCA device data showed a significant difference between the groups in terms of total demand at the 24 th hour postoperatively, (group M-TAPA, *n* = 25.5 (0–195), group control, *n* = 47 (11–264); *p* = 0.001).

## Discussion

We investigated the effects of M-TAPA block on postoperative respiratory functions in patients with obesity who underwent LSG. We observed that postoperative spirometric pulmonary function test results were less affected in the group M-TAPA compared to the group Control. We found that the patients in the M-TAPA group consumed fewer opioids and had higher QoR-15 scores in the postoperative 24 h compared to the control group. Additionally, both resting and dynamic NRS pain scores were higher in the group control.

Incisional pain after abdominal surgery causes a reflex increase in muscle tone, which reduces the patient’s depth of inspiration and causes shallow breathing. As a result, tidal volume, vital capacity, and functional residual capacity decrease [8]. A key factor in reducing postoperative pulmonary complications is effective pain management [9].

In a normal respiratory cycle, intercostal muscle activity increases during inspiration and abdominal muscle activity increases during expiration, and the abdominal muscles contribute to the movement of the diaphragm towards the rib cage. After abdominal surgery, the expiratory pattern is severely affected due to both muscle incision and painful sensation [4]. The PEF is the highest flow rate that a person can achieve at the beginning of forced expiration and is effort-dependent. It reflects the power of the expiratory muscles [10]. In the present study, we found that PEF values were significantly higher in the group M-TAPA at the postoperative 1 st hour and higher than the group control at all other time points. We consider that this result is due to the fact that M-TAPA block provides effective analgesia and patients can use their expiratory muscles more effectively.

Restrictive ventilatory dysfunction develops following abdominal surgery. In restrictive disorders, FEV1 and FVC decrease, and FEV1/FVC, a ratiometric value, usually does not change [4, 11]. The postoperative FEV1 and FVC values decreased in all patients in our study, and the FEV1/FVC values were above 70% in all-time point measurements. These results indicate that obstructive ventilatory dysfunction was not present in the patients.

The FEV1 value is effort-dependent, and 70–80% of the vital capacity is exhaled in the first second in healthy individuals [12]. The severity of restrictive ventilatory dysfunction is recommended to be determined based on FEV1. A predicted FEV1 value > 70% was defined as mild, 60–69% as moderate, 50–59% as moderate-severe, 35–49% as severe, and < 35% as very severe impairment [13]. In our study, the severity of impairment was moderate in the group M-TAPA and severe in the group control according to the measurements at the postoperative first hour. In the measurements at all other time points, it was observed that the values in the group M-TAPA were higher. This indicates that the M-TAPA block also reduces the severity of restrictive impairment after metabolic bariatric surgery.

Aboseif et al. [14] investigated the effects of transversus abdominis plane (TAP) block on postoperative lung function in LSG and performed spirometry in patients preoperatively and at the 24 th hour postoperatively. They reported that preoperative and postoperative FVC, FEV1, and FEV1/FVC values were similar between the TAP block and the control group. However, they indicated that the values were less affected in the TAP block group compared to the control group. In their study, it was observed that there was no difference between the groups in terms of pain scores at the 24 th hour. In our study, there was a significant difference between the groups in terms of spirometric values at the 1 st postoperative hour. At the 24 th hour, spirometric values were similar between the groups. In the present study, there was a significant difference in NRS scores between the groups at the first hour, but there was no difference at the 24 th hour. These results lead us to consider that NRS scores have a direct effect on spirometric values.

The risk of postoperative pulmonary complications is higher in patients with obesity than in patients without obesity, and hypoxia and atelectasis are more common. Most patients with obesity also suffer from obstructive sleep apnea and obese hypoventilation syndrome [3]. Pain is very common following metabolic bariatric surgery. Parietal pain due to abdominal wall damage during trocar insertion is responsible for 50–70% of the pain felt after laparoscopic metabolic bariatric surgery [15]. In the management of pain in these patients, opioids with many side effects are generally administered. The respiratory side effects of opioids, which are among the most undesirable side effects of opioids, are dose-dependent [16]. In our study, we performed an M-TAPA block as part of a multimodal analgesia regimen. This block has been shown to affect the lateral and anterior branches of the thoracoabdominal nerves between T6 and T11 [17]. Postoperative pain palliation and minimizing opioid consumption were aimed at the block application. In the present study, it was found that the patients in the M-TAPA group required less opioids in the postoperative 24 h compared to the control group.

Turunc et al. [18] compared the effects of M-TAPA block and external oblique intercostal block on postoperative opioid consumption, pain scores, and quality of recovery in LSG. In their study, they reported that the median NRS pain score of patients who underwent M-TAPA block was < 3 in the first 24 h postoperatively. They concluded that the M-TAPA block contributed positively to postoperative pain scores and recovery quality. In our study, we found that the postoperative NRS pain scores of the patients in the group M-TAPA were < 3, and significantly lower in the first 12 h compared to the group control.

The QoR-15 questionnaire focuses on patients’ comfort and pain, measures the quality of patient’s recovery, and is one of the important outcomes evaluated in clinical trials of surgical patients [19]. In a case series including 12 patients undergoing LSG, M-TAPA block was applied to the patients, and the quality of recovery was evaluated with the QoR-15 questionnaire [7]. They stated that the M-TAPA block can provide a satisfactory quality of recovery in LSG. The pain subdimension questions in this questionnaire are responded to based on the pain experienced in the last 24 h. In our study, the score of the pain subdimension in the QoR-15 questionnaire repeated at the 24 th hour postoperatively in the group M-TAPA was higher than in the group control. We think that this result was also contributed by the M-TAPA group’s better handle on the drain. According to this questionnaire, which measures the pain intensity of the postoperative 24-h period, it is seen that the M-TAPA group patients spent this period in less pain than the group control. We think that this condition affected the scores of other subdimensions so that the global score of the group M-TAPA was higher.

### Limitations

Our study has some limitations. The first of these is that we stopped evaluating respiratory functions after the 24 th hour. We did not evaluate how long respiratory dysfunction lasted in our patients, which could last up to 10 days after surgery, and on what day it returned to baseline values. Another limitation is that we did not evaluate the dermatome range covered since the blocks were applied while the patients were intubated. The lack of sham M-TAPA block application to the control group is also among our limitations. Finally, we did not evaluate the effects of the block on QoR-15 scores in the long term, such as after discharge.

## Conclusion

We determined that the M-TAPA block applied to patients with obesity who underwent LSG reduced postoperative respiratory dysfunction and the amount of opioids required. We observed that the M-TAPA block improved patients’ comfort in the first 24 h postoperatively. The M-TAPA block may be considered for inclusion in the multimodal analgesia regimen for patients undergoing LSG.

## Data Availability

No datasets were generated or analysed during the current study.
